# TRIM3 Regulates the Motility of the Kinesin Motor Protein KIF21B

**DOI:** 10.1371/journal.pone.0075603

**Published:** 2013-09-24

**Authors:** Dorthe Labonté, Edda Thies, Yvonne Pechmann, Alexander J. Groffen, Matthijs Verhage, August B. Smit, Ronald E. van Kesteren, Matthias Kneussel

**Affiliations:** 1 Department of Molecular Neurogenetics, Center for Molecular Neurobiology, ZMNH, University Medical Center Hamburg-Eppendorf, Hamburg, Germany; 2 Department of Molecular and Cellular Neurobiology, Center for Neurogenomics and Cognitive Research, VU University, Amsterdam, Netherlands; Stanford University School of Medicine, United States of America

## Abstract

Kinesin superfamily proteins (KIFs) are molecular motors that transport cellular cargo along the microtubule cytoskeleton. KIF21B is a neuronal kinesin that is highly enriched in dendrites. The regulation and specificity of microtubule transport involves the binding of motors to individual cargo adapters and accessory proteins. Moreover, posttranslational modifications of either the motor protein, their cargos or tubulin regulate motility, cargo recognition and the binding or unloading of cargos. Here we show that the ubiquitin E3 ligase TRIM3, also known as BERP, interacts with KIF21B via its RBCC domain. TRIM3 is found at intracellular and Golgi-derived vesicles and co-localizes with the KIF21B motor in neurons. *Trim3* gene deletion in mice and TRIM3 overexpression in cultured neurons both suggested that the E3-ligase function of TRIM3 is not involved in KIF21B degradation, however TRIM3 depletion reduces the motility of the motor. Together, our data suggest that TRIM3 is a regulator in the modulation of KIF21B motor function.

## Introduction

Eukaryotic cells depend on active cytoskeleton transport to navigate intracellular cargo towards specific subcellular domains. Due to their distinct morphological and functional polarity neurons sort and transport proteins, vesicles or mRNA-protein granules either to the axonal or somato-dendritic compartment. Kinesin, dynein or myosin superfamily proteins mediate active transport along microtubules or actin filaments, respectively [1]. Motor proteins act in concert with cargo adapters that mediate transport specificity and encode steering and directionality of individual cargos to their functional destinations [2,3]. Furthermore, posttranslational modifications (PTMs) of tubulin, the core proteins of microtubules, regulate neuronal transport processes. These PTMs include phosphorylation, de-tyrosination, acetylation and polyglutamylation [4,5] and are thought to alter transport parameters by regulating the interaction of microtubules with associated proteins and/or motor-cargo-complexes [6-8]. In addition, PTMs of motor proteins have been shown to regulate cargo unloading and the activation of auto-inhibited protein conformations [9,10].

The kinesin superfamily protein KIF21B is encoded by one of 45 KIF genes identified in the human and murine genome [1]. It is exclusively expressed in spleen and brain tissues, is enriched in neurons and mainly localizes to dendrites [11]. The N-terminal motor domain of KIF21B moves on microtubule tracks in a plus-end directed manner. Due to the polarity of microtubules in distal dendrites [12] KIF21B is suggested to mediate anterograde long-distance transport to the cell periphery, however, up to date no cargo of KIF21B motors has been identified. Notably, recent studies reported SNPs within or close to the *KIF21B* gene as susceptibility locus for the inflammatory diseases multiple sclerosis, Crohn’s disease and ankylosing spondylitis [13-16].

TRIM3 (*tripartite-motif containing 3*) is a member of the TRIM protein superfamily, which represents the largest class of single RING-finger E3 ligases in mammals, comprising more than 70 members that are involved in a variety of cellular processes [17]. E3 ubiquitin ligase activity has been demonstrated for different TRIM-family members [18,19], including TRIM3 [20]. Some TRIM-proteins may however also act as SUMO E3 ligases [21]. TRIM3 is highly enriched in neurons and is suggested to mediate polyubiquitination and subsequent proteasomal degradation of the postsynaptic scaffold protein GKAP [20]. Later studies however failed to connect TRIM3 to the CaMKII-dependent removal of synaptic GKAP in processes of activity-dependent homeostatic scaling [22]. TRIM3 also binds the actin-based motor protein myosin V [23] and the actin-binding protein alpha-actinin-4 [24]. Both factors participate together with TRIM3 in the CART-complex (cytoskeleton-associated-recycling or transport complex) that regulates vesicular receptor recycling in non-neuronal cells [25]. Finally, TRIM3 depletion is suggested to account for decreased GABA_A_-receptor cell surface levels in cultured cortical neurons [26]. Together, these data suggest independent roles for TRIM3 in different pathways, however a functional role of TRIM3 in neuronal trafficking is currently not described.

Here, we show that KIF21B directly binds to and colocalizes with TRIM3 in neurons. Studies using *Trim3* knockout neurons and TRIM3-overexpressing neurons reveal that TRIM3 is not involved in KIF21B degradation, which might depend on polyubiquitination, however affects the functional motility of the motor protein.

## Results

### TRIM3 directly binds to and colocalizes with the microtubule-dependent motor protein KIF21B

**Figure 1:**
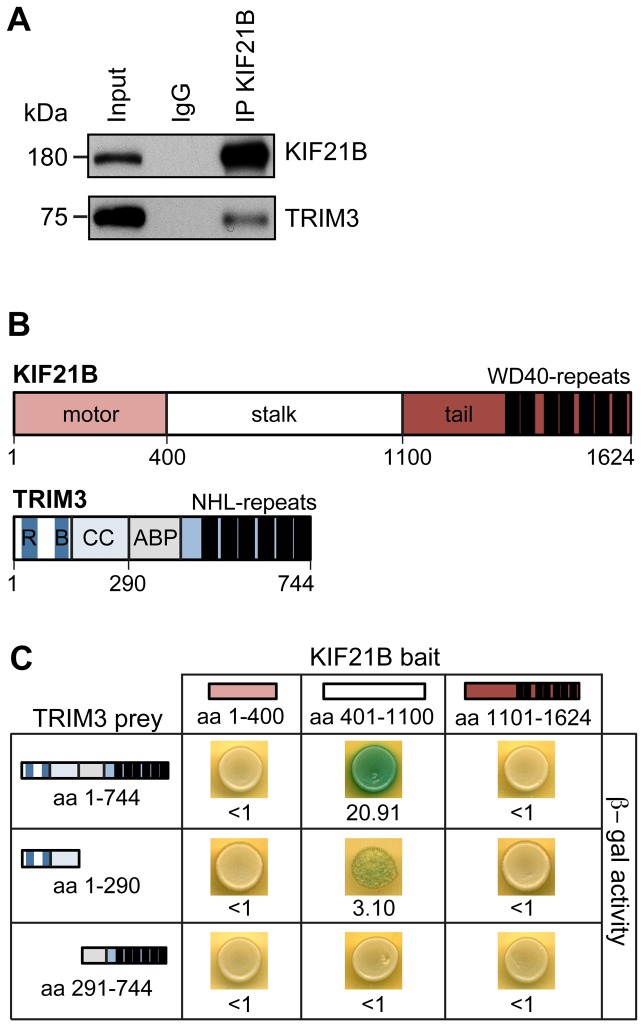
Interaction of KIF21B and TRIM3 in vitro. (A) Co-immunoprecipitation: a KIF21B-specific antibody precipitates endogenous KIF21B and co-precipitates endogenous TRIM3 from brain lysate indicating *in* *vitro* binding of both proteins. (B) Schematic representation of the domain structures of KIF21B and TRIM3. WD40-repeats: tryptophan-aspartic acid (W-D) dipeptide repeats; R: RING; B:B-box; CC: Coiled-coil; ABP: ABP (actin-binding protein)-like domain; NHL: NCL-1/HT2A/Lin-41. (C) Mapping of interaction domains using the DupLEX-A yeast two-hybrid-system. Full-length TRIM3 and the TRIM3-RBCC-domain (aa1-290) bind the stalk region of the motor protein KIF21B. Beta-galactosidase activity (blue signals). Values represent average signal intensities (arbitrary units).

Using co-immunoprecipitation experiments (co-IPs) with a KIF21B-specific antibody from rat brain lysate enriched for intracellular vesicles and protein complexes (P4 lysate, 400,000 x g), we identified interaction of KIF21B with the RING-finger E3 ligase TRIM3 (Figure 1A). To investigate whether both proteins interacted directly, and to map binding domains, we applied the lacZ-reporter gene assay of the DupLex-A yeast-two-hybrid system. KIF21B-bait constructs of either the motor-domain (aa 1-400), the stalk-domain (aa 401-1100) or the tail-domain (aa 1101-1624) were used in combination with TRIM3-prey constructs, encoding either full length TRIM3 (aa 1-744), only the N-terminal Ring-B-box-Coiled-Coil (RBCC) domain (aa 1-290) or only the C-terminal actin-binding-protein-like (ABP) and NHL repeat domains (aa 291-744) (Figure 1B). We found that TRIM3, in particular its N-terminal RBCC domain (aa 1-290) known to be involved in substrate binding and ligase function [27], directly binds the internal stalk-domain of KIF21B (Figure 1C), suggesting that KIF21B may be a substrate of TRIM3.

**Figure 2:**
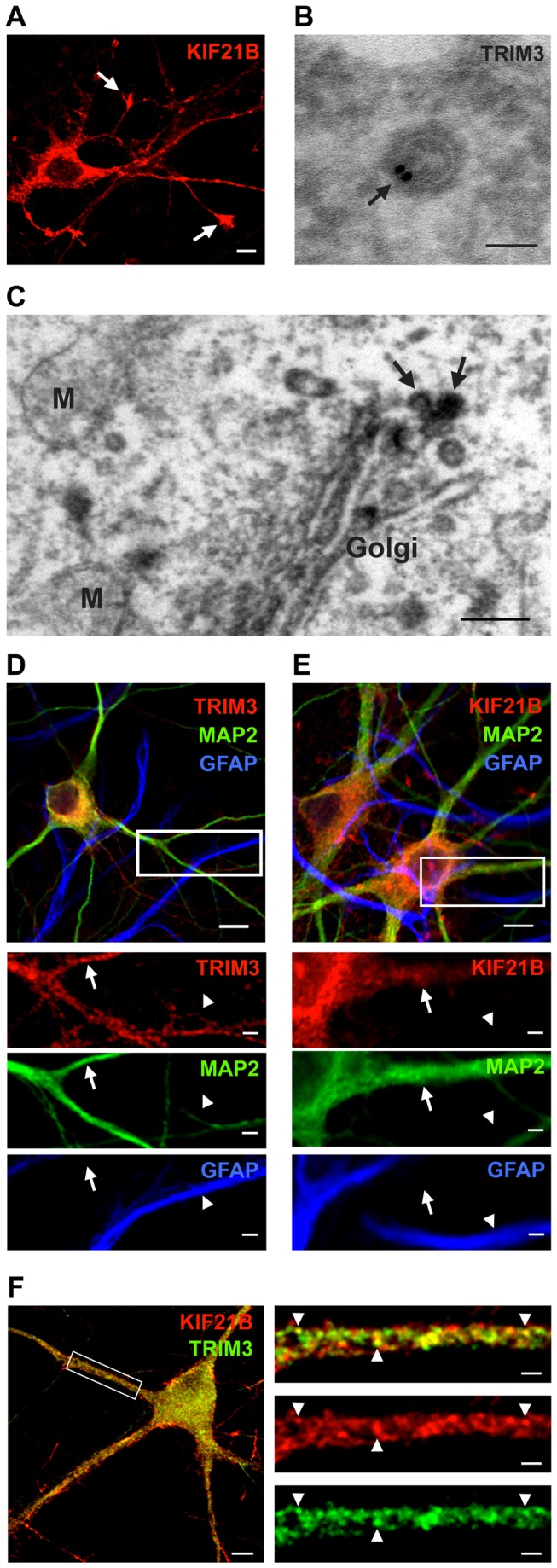
Subcellular localization of KIF21B and TRIM3. (A) KIF21B locates to the somato-dendritic compartment of neurons and is prominent in growth cones of young neurons (DIV7) (arrows). (Scale bar: 20 µm.) (B) Electron microscopy analysis, immunogold signals. TRIM3 locates close to the membrane of putative transport vesicles in neurons derived from hippocampal slices (arrow) (Scale bar: 50 nm.). (C) Electron microscopy analysis, DAB signals. TRIM3 locates to vesicles at the Golgi apparatus (arrows). M: mitochondria, Golgi: Golgi apparatus (Scale bar: 200 nm). (D, E) TRIM3 (red, left) or KIF21B (red, right) colocalize with MAP2-positive dendrites (green, arrows) but are not detected in GFAP-positive astrocytes (blue, arrowheads). (Scale bars: 20 µm.) (F) KIF21B and TRIM3 colocalize in punctate structures (yellow, arrowheads) across neuronal dendrites. (Scale bar: 20 µm, scale bar boxed region: 5 µm.).

KIF21B is mainly expressed in spleen and brain. Brain expression is predominantly neuronal, with strong enrichment in the somato-dendritic compartment [11]. Accordingly, we detected KIF21B signals both in the soma and throughout the neurites by immunostaining of mouse hippocampal neurons cultured for 7 days in vitro (DIV7) (Figure 2A). Notably, KIF21B was highly enriched in growth cones located at neurite tips (Figure 2A, arrows). In control experiments using shRNA-mediated knockdown of mouse KIF21B gene expression, the KIF21B antibody was shown to specifically detect the KIF21B protein (Figure S1A).

TRIM3 has been implicated in myosin Va- and myosin Vb-mediated vesicle trafficking [23]. We therefore asked whether neuronal TRIM3 would in general locate to vesicular structures in neurons. Hippocampal sections derived from adult mice were immunolabeled with a TRIM3-specific antibody and subsequently processed for electron microscopy (EM). At the ultrastructural level, we found TRIM3 immunogold signals close to the membrane of putative transport vesicles (Figure 2B), the latter with a typical size range from 50-100 nm [28]. Consistently, immunoperoxidase labeling with DAB also revealed TRIM3-positive vesicles budding from the Golgi apparatus (Figure 2C). Confocal microscopy further revealed the presence of TRIM3 and KIF21B in MAP2-positive neuronal dendrites (Figure 2D and 2E, arrows), whereas GFAP-positive astrocytes lack TRIM3 and KIF21B signals (Figure 2D and 2E, arrowheads). Consistent with the observed *in vitro* binding of KIF21B and TRIM3, endogenous signals of both proteins were found to colocalize in dendrites of DIV21 cultured hippocampal neurons (Figure 2F, arrowheads, Figure S1B).

### Generation of a *Trim3*-knockout-mouse

**Figure 3:**
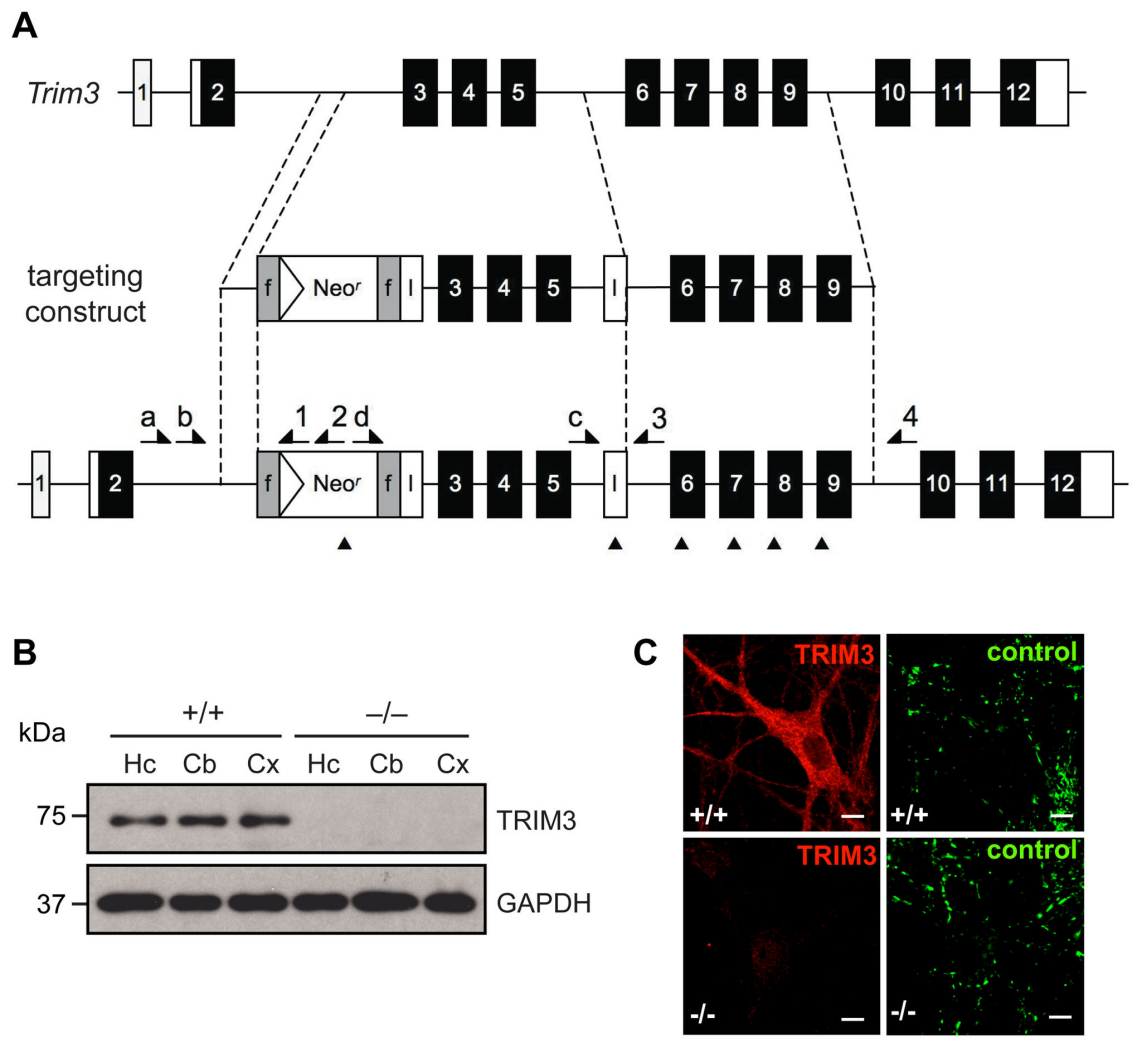
Generation of a *Trim3* knockout mouse and validation of TRIM3 depletion. (A) Schematic representation of the *Trim3* gene and the *Trim3* targeting construct. Homologous recombination of the targeting construct produces a mutant *Trim3* gene containing a Neo^r^ cassette flanked by frt sites (grey boxes, f) and loxP sites (white boxes, l) flanking exons 3-5. Recombination of the loxP sites results in excision of exons 3-5; recombination of the frt sites results in excision of the Neo^r^ cassette. Forward primers (a-d), reverse primers (1-4) and BglII restriction sites (▲) used for genotyping are indicated (see Figure S1C). (B) Western blotting confirms the absence of TRIM3 in hippocampus (Hc), cerebellum (Cb) and cortex (Cx). (C) Immunocytochemistry confirms the absence of TRIM3 in cultured hippocampal neurons derived from *Trim3* knockout mice (DIV12). Control: Synaptic vesicle protein SV2. (Scale bars: 20 µm.).

To investigate the role of TRIM3 in KIF21B trafficking we established a *Trim3*-knockout mouse. A conditional gene targeting vector containing loxP sites to remove exons 3 to 5 was used for homologous recombination in E14.1 embryonic stem cells (Figure 3A). Removal of exons 3-5 deletes amino acids 45-232 of the TRIM3 protein, (corresponding to the C-terminal half of the RING-domain, the complete B-box and most of the coiled-coil domain) and causes a frame shift leading to a premature stop codon. The neomycin-selection cassette, flanked by frt-sites, was removed through crossbreeding with a flp-deleter mouse line [29]. TRIM3 depletion was induced by crossbreeding with a Cre-deleter mouse [30].

The correct integration of the targeting construct was verified by long-range genomic PCR in combination with restriction analysis using diagnostic restriction sites that were introduced with the targeting vector (Figure 3A, Figure S1C). In addition, Western blot analysis with a TRIM3-specific antibody confirmed that TRIM3 protein levels were lost in -/- animals, as compared to +/+ genotypes (Figure 3B). Accordingly, immunocytochemistry revealed a loss of TRIM3 signals in cultured hippocampal neurons derived from -/- mice, as compared to +/+ littermate controls (Figure 3C). *Trim3*-knockout mice were viable, fertile and showed no obvious morphological abnormalities.

### The E3 ligase function of TRIM3 does not regulate KIF21B degradation

**Figure 4:**
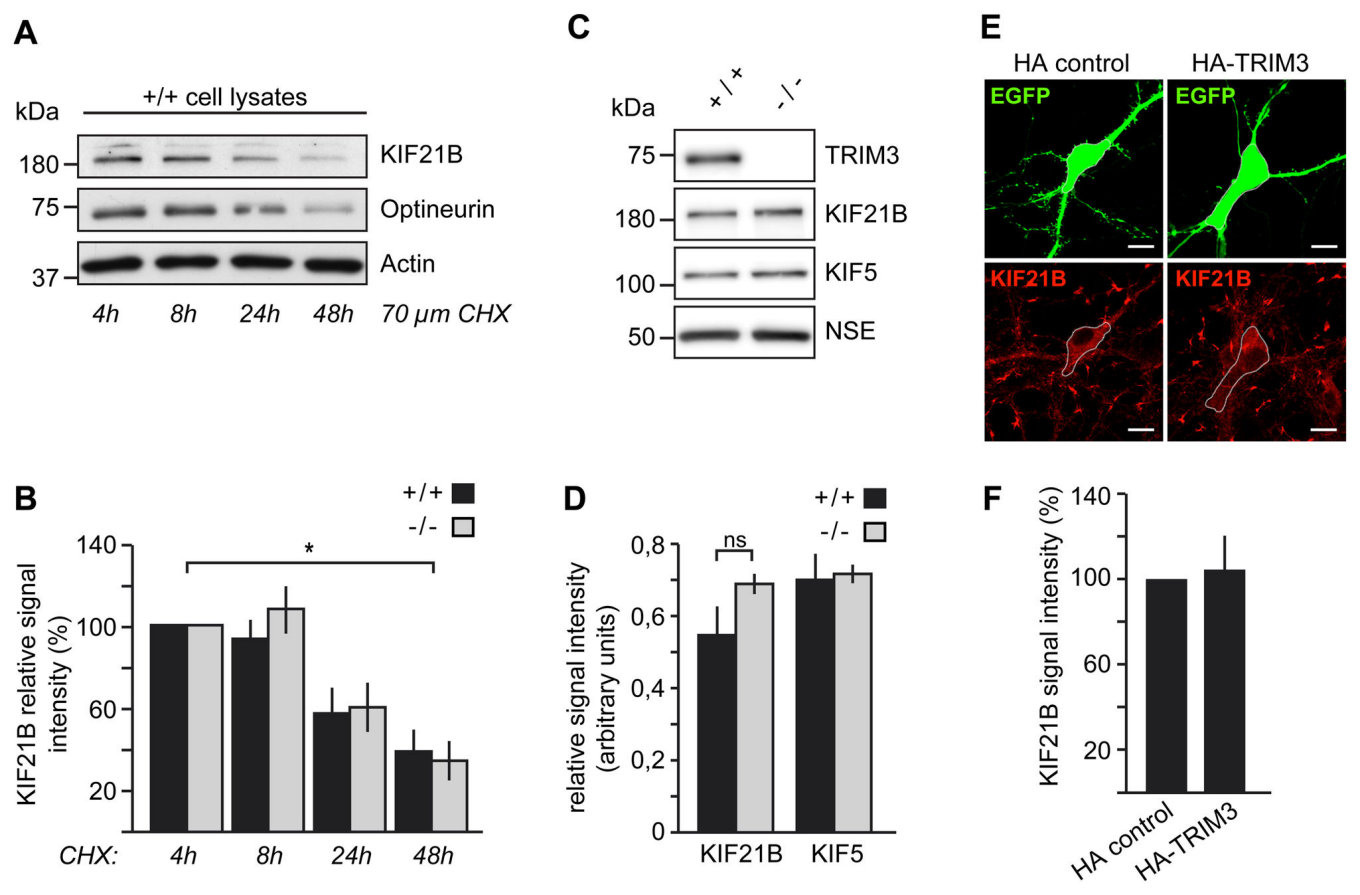
The E3 ligase TRIM3 is not involved in the degradation of the motor protein KIF21B. (A, B) KIF21B half life analysis using cycloheximide (CHX) chase experiments. More than 50% of KIF21B degrades within 48 hours in cultured hippocampal neurons (DIV16) derived from wildtype (+/+) mice. TRIM3 genetic depletion does not alter the half life of KIF21B, as assessed through evaluation of relative KIF21B signal intensities. Optineurin and actin served as controls. Relative signal intensity of KIF21B/actin ratios in %. n=3, each. 4h: wildtype (+/+) 100%, knockout (-/-) 100%; 8h: wildtype (+/+) 93.9±9.2, knockout (-/-) 110.4±12.9; 24h: wildtype (+/+) 57.8±11.5, knockout (-/-) 61.5±12.0; 48h: wildtype (+/+) 39.4±10.3, knockout (-/-) 35.4±9.6. (C, D) Relative signal intensities of KIF21B and KIF5 in hippocampal lysates remain equal across the genotypes (wildtype (+/+) versus *Trim3* knockout (-/-)). Relative signal intensity of KIF/NSE ratios in %. n=4 each. KIF21B: wildtype (+/+) 0.55±0.08, knockout (-/-) 0.69±0.03; KIF5: wildtype (+/+) 0.70±0.07, knockout (-/-): 0.72±0.03. ns: not significant. (E, F) Overexpression of TRIM3 does not alter endogenous KIF21B protein levels. Cultured hippocampal neurons (DIV10) were transfected with vectors encoding HA-TRIM3 or HA, respectively. Coexpression of GFP served as transfection control and volume marker. Cells were fixed and stained for endogenous KIF21B at DIV14. Somatic KIF21B signal intensity: HA-control: set to 100%, n= 40; HA-TRIM3: 104±17%, n=38. (Scale bars in E: 20 µm.) Data: means±SEM.

TRIM3 has been shown to be a functional E3 ubiquitin ligase [20]. We therefore asked whether TRIM3 mediated degradation of the KIF21B motor protein. To assess the half life of KIF21B we performed a cycloheximide (CHX) chase experiment in cultured wildtype hippocampal neurons. Cells were treated with the translational inhibitor CHX for 4-48 hours. Because of their established half lives, immunodetection of optineurin and actin served as positive controls (optineurin t_½_ < 48 h [31],; actin t_½_ > 48h [32],). Quantitative analysis revealed no KIF21B degradation within the first 8 h after CHX application (Figure 4A), indicating that the motor protein is comparatively stable. Thereafter however, KIF21B is substantially degraded, with a half life in the range of 24-48 h (Figure 4A).

Next, we compared KIF21B stability in hippocampal neurons derived from either wildtype (+/+) or TRIM3-deficient (-/-) mice. Notably, we observed no difference between the genotypes (Figure 4B), indicating that the absence of TRIM3 does not alter KIF21B levels over time. This result was consistent with the fact that equal amounts of KIF21B were obtained from P15 hippocampal lysates (S1, 1,000 x g) derived from both genotypes (Figure 4C, D). Also the expression levels of the kinesin-family motor KIF5, previously reported to associate with TRIM3 in RNA-transporting granules [33], did not differ between wildtype (+/+) and *Trim3* knockout (-/-) lysates, as compared to a loading control (NSE) (Figure 4C, D).

To rule out compensation of TRIM3 deficiency through other TRIM family proteins, we also tested whether TRIM3 overexpression might decrease KIF21B protein stability. Neuronal overexpression of HA-TRIM3 did not alter the relative signal intensities of endogenous KIF21B (Figure 4E, F). We therefore conclude that TRIM3 is not a regulator of KIF21B protein degradation.

### TRIM3 modulates KIF21B transport

**Figure 5:**
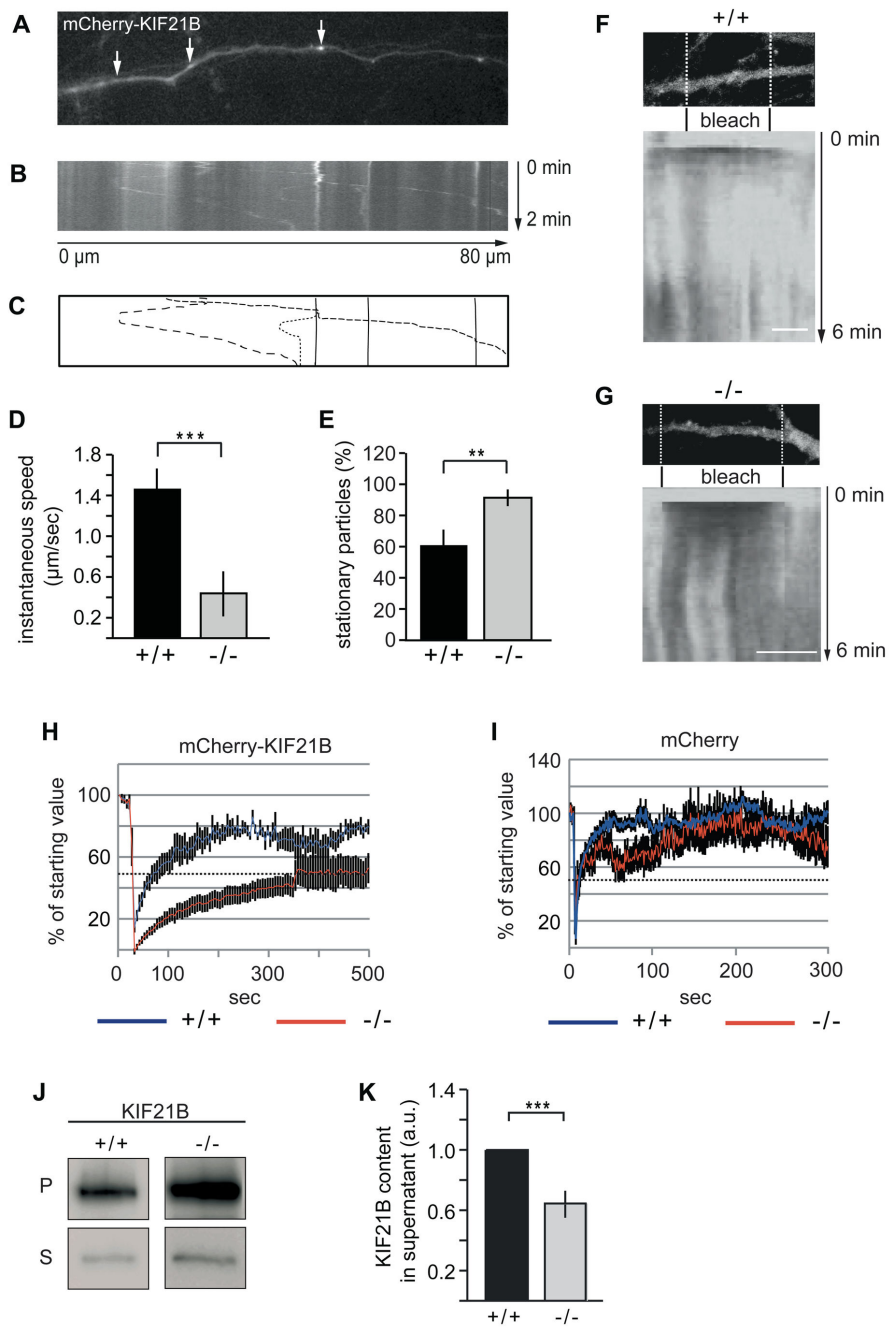
Analysis of mCherry-KIF21B mobility in wildtype (+/+) and *Trim3* knockout (-/-) neurons. (A-E) Cultured hippocampal neurons at DIV6-11. (A, B) Particles in wildtype (+/+) neurons were analysed with an image acquisition rate: 1 image/sec. The corresponding kymograph represents a region of 80µm. (C) Schematic representation of mobile (arrows in A) and stationary particles. (D) Quantitative evaluation of instantaneous particle speed across the genotypes (+/+: 1.47+0.20 µm/sec; -/-: 0.44+0.22 µm/sec). (E) Quantitative evaluation of the number of stationary particles across the genotypes (+/+: 60.90+9.76%; -/-: 91.40+3.90%). (F-I) FRAP-analysis of mCherry-KIF21B in cultured hippocampal neurons (DIV11) derived from wildtype (+/+) or *Trim3* knockout (-/-) mice. After photobleaching of transfected cultured neurons, the recovery of mCherry-KIF21B fluorescent signals was subsequently analysed over 500 sec with an image acquisition rate of 1 image/5 sec. mCherry was analysed with an image acquisition rate of 1 image/sec (control). (F, G) mCherry-KIF21B in wildtype (+/+) and in *Trim3* knockout (-/-) neurons. Scale bars: 10 µm. Kymographs display the bleached regions and the recovery of fluorescence over 6 min. (H) Quantification of mCherry-KIF21B fluorescent recovery in wildtype (+/+) and *Trim3* knockout (-/-) neurons. The recovery rate is much faster in wildtype (+/+) neurons, as compared to *Trim3* knockout (-/-) neurons. (I) Control: quantification of mCherry fluorescent recovery in wildtype (+/+) and *Trim3* knockout (-/-) neurons reveals equal values. (J, K) Analysis of KIF21B content in pellet and supernatant fractions by Triton-X-100 extraction. Pellet (P) and supernatant (S) fractions derived from cultured hippocampal wildtype (+/+) or *Trim3* knockout (-/-) neurons (DIV20-23) were subjected to Western blot analysis. Quantification of KIF21B content in supernatant: wildtype (+/+) values set to 1 (3 individual experiments with a total of n=10), *Trim3* knockout (-/-)= 0.65±0.08 (3 individual experiments with a total of n=8). (a.u.= arbitrary units.) Data: means±SEM.

Ubiquitination is not necessarily involved in protein degradation, and different non-degradative functions of ubiquitin and its ligases have been reported. For instance, ubiquitination also serves as a post-translational modification that creates versatility in cell signalling [34]. To check whether TRIM3 deficiency changes KIF21B function, we analysed the mobility of this motor protein using neuronal live cell imaging through time-lapse video microscopy. Upon expression of a mCherry-KIF21B fusion protein, we identified mobile fluorescent particles in neuronal dendrites derived from both genotypes (Figure 5A-C, Figure S1D-F, movie S1). The mCherry-KIF21B fusion proteins displayed directed long-distance mobility over several µm with velocities typical for kinesin-mediated transport [35]. However, in the absence of *Trim3* gene expression, mCherry-KIF21B particle speed was significantly reduced (Figure 5D). While mCherry-KIF21B moved in both anterograde and retrograde directions in wild type neurons (n=33 particles), we did not observe anterograde mCherry-KIF21B movement in neurons derived from *Trim3* knockout mice (n=19 particles). Consistent with these differences, the number of stationary particles was significantly increased in knockout neurons (Figure 5E), suggesting that TRIM3 critically regulates motor function and might affect directionality of KIF21B, which typically represents a plus end-directed motor with a preference for anterograde movement in distal dendrites [11].

We next applied fluorescence recovery after photobleaching (FRAP imaging) to further evaluate the mobility of mCherry-KIF21B in neurons derived from both genotypes (Figure 5F-I). Dendritic regions of interest (ROIs) of 20 µm each were photobleached from neurons, expressing mCherry-KIF21B, while mCherry expression served as a control. Notably, the kinetics of mCherry-KIF21B mobility differed significantly between genotypes (Figure 5H). In dendrites of wildtype (+/+) neurons mCherry-KIF21B fluorescence recovered with a coefficient of 3 µm^2^/sec and a half time recovery rate of t^1/2^=55 sec (n=9, Figure 5H). In contrast, recovery of mCherry-KIF21B fluorescence in dendrites of *Trim3* knockout (-/-) neurons was much slower with a coefficient of 0.3 µm^2^/sec and a half time recovery rate of t^1/2^=395 sec (n=18, Figure 5H). Also, the maximum percentage of recovery was found to be lower in dendrites of *Trim3* knockout (-/-) neurons, suggesting that more motors might be bound to their tracks but remain stationary when TRIM3 is absent (compare with Figure 5E). Control experiments performed with mCherry showed no differences between the genotypes (coefficient: 9µm^2^/sec; half time recovery rate: t^1/2^=12 sec; n=5, each) (Figure 5I). To further investigate motor-track binding, we applied a cell extraction assay to separate cytoskeleton-attached motor proteins (pellet) from unbound motors (supernatant) (Figure 5J, K). Notably this assay identified significantly less KIF21B in supernatants derived from *Trim3* knockout (-/-) neurons, suggesting that the absence of TRIM3 promotes the affinity between the motor and its track.

Together, TRIM3 positively affects KIF21B motor motility, whereas genetic depletion of TRIM3 expression slows down the motor protein and may affect cytoskeletal attachment of KIF21B.

## Discussion

In this study we identified interaction of TRIM3 with the microtubule-dependent motor protein KIF21B. Studies with neurons obtained from *Trim3* knockout mice suggest that TRIM3 is not involved in KIF21B degradation, but regulates the motility of KIF21B. In particular, TRIM3 depletion affects anterograde-directed movement of KIF21B and significantly slows down motor speed.

A testis-specific TRIM family protein TRIM60/RNF33 was recently found to also interact with microtubule motors, namely KIF3A and KIF3B. Similar as TRIM3, TRIM60 binds to the stalk or rod domain of the kinesin and TRIM60-KIF3 interactions do not require the adaptor kinesin-associated protein KAP3, which regulates motor-cargo association [36]. TRIM3 also binds directly to KIF21B, independent of any cargo adapter, suggesting that TRIM3 modulates KIF21B motor function instead of being transported by this motor.

At the subcellular level, we identified TRIM3 in a punctate manner throughout somata, dendrites and axons. Previous studies have reported that TRIM3, when lacking its C-terminal myosin-binding domain, blocks NGF-induced neurite outgrowth in PC12 cells [23]. TRIM3 might therefore be critical in organelle transport which amongst others serves neurite outgrowth. A role for TRIM3 in trafficking was also suggested in HeLa cells. TRIM3 is part of the CART complex, which associates with recycling endosomes, the actin filament motor proteins myosin V, and the actin-associated protein alpha-actinin-4 [25]. CART is required for efficient endosomal recycling of the transferrin receptor (TfR) back to the plasma membrane, and was suggested to play a role in constitutive receptor recycling in general. With respect to TRIM3 being a potential regulator of motor protein trafficking, it is interesting to note that GABA_A_ receptor (GABA_A_R) cell surface levels and the amplitude of miniature inhibitory postsynaptic currents (mIPSCs) are reduced in cortical neurons derived from TRIM3 depleted mice [26]. However, whether TRIM3’s role in the CART complex is ubiquitin-independent or involves its ubiquitin E3 ligase function is currently unknown and requires further investigation.

Monoubiquitination has been implicated in endocytosis of cell surface proteins, a process that employs both actin filament-based and microtubule-based motor proteins [1,37]. Mosesson and coworkers reported that monoubiquitination regulates the endosomal localization of Lst2, a negative regulator of EGF receptor signaling. Lst2 also binds TRIM3, and even though TRIM3 does not alter the ubiquitination state of Lst2, it is thought to regulate the subsequent recycling of Lst2-containing complexes [38].

Despite a prominent role of myosin motors in endocytic recycling, kinesins also have been implicated in receptor recycling processes. For instance, KIF16B transports early endosomes to the plus end of microtubules and overexpression of this motor protein relocates early endosomes to the cell periphery [39]. In general, endocytosis and recycling are functionally connected two-step processes that involve both the submembrane actin- and the microtubule cytoskeleton. In receptor internalization, actin-based motors act upstream of microtubule motors and vesicular cargo is transferred between the different cytoskeletal tracks. Candidate factors to regulate this transition of motors and tracks involve the GABA_A_R binding protein muskelin [37]. Whether TRIM3’s interaction with myosin V and KIF21B points to a similar role at the actin-microtubule interface is currently unclear. It is presently also not known whether KIF21B acts in receptor recycling, however KIF21B/TRIM3 colocalized puncta are found in proximity to the plasma membrane and KIF21B motors are therefore candidate players in the transport of TRIM3-regulated cell surface proteins such as postsynaptic GABA_A_Rs [26].

The motility of kinesins is thought to be regulated through conformational changes and through binding of accessory proteins. Kinesin-1 is proposed to switch between a folded inactive state and an open active state [40], a process that seems to involve Sunday driver/JIP3 (also known as syd or JSAP1), which enhances kinesin motility in an *in vitro* assay. Sunday driver binding to the kinesin heavy chain (KHC) tail is suggested to relieve the inhibition of the tail domain, thereby activating or opening KHC to bind microtubules for long-range motility [41]. Other factors that positively regulate kinesin motility include *Drosophila* Pat1 that transports *oskar* mRNA in *Drosophila* oocytes [42] and the Ran binding protein 2 (RanBP2), which activates the ATPase activity of KHC, thereby potentially regulating kinesin velocity and processivity [43]. Neuronal proteins that regulate the transport direction include huntingtin (htt). Upon Akt kinase-dependent phosphorylation of serine 421, htt recruits kinesin-1 to the dynactin complex on vesicles and microtubules, thereby promoting anterograde transport. Conversely, when htt is not phosphorylated, kinesin-1 detaches and vesicles are more likely to undergo retrograde transport [44].

Our data suggest that TRIM3 is another player in positively modulating anterograde kinesin motility. They provide a starting point to further investigate the underlying mechanism, possibly by addition of a PTM to KIF21B or by switching the conformation of the motor protein. Alternatively, TRIM3 might ubiquitinate an unknown target that indirectly affects KIF21B motility. Together, the present results provide another example that TRIM superfamily proteins can mediate regulatory functions in protein trafficking.

## Methods

### Ethics Statement

All animal work has been conducted according to the relevant national and international guidelines. The generation of *Trim3* knockout mice was approved by the Animal Welfare Committee (DEC) of the VU Amsterdam (protocol FGA 03-02). Mice were sacrificed by decapitation. The sacrifice of mice in this study was approved by the VU Amsterdam animal welfare (DEC) committee (protocol FGA 03-02). In addition, mice were sacrificed in the Hamburg laboratory by decapitation to collect brains for the generation of neuronal tissue cultures. This method was approved by the following number: TierSchG §4, UKE ORG 401.

### Yeast Two-Hybrid Screening

The DupLEX-A yeast two-hybrid system (Origene, Rockville, MD) was used for protein-protein interaction screening. Interaction of KIF21B-bait constructs (pGilda-vector) and TRIM3-prey constructs (pJG-4-5-vector) were assayed by activation of the lacZ reporter. -galactosidase activity was quantified as the average signal intensity of individual colonies (arbitrary units) with Metamorph software (Molecular Devices, Sunnyvale, CA).

### Constructs

Yeast-Two-Hybrid-Screening: to generate bait constructs KIF21B-subdomains (motor: aa 1-400, stalk: aa 401-1100, tail: aa 1101-1624) were amplified from a cDNA-clone containing murine KIF21B-ORF (Origene, Rockville, MD) and inserted into pGILDA-vector as *Eco*RI-*Sal*I fragments. To generate prey constructs full-length TRIM3 and TRIM3 subdomains were amplified from a cDNA-clone containing murine TRIM3-ORF (generated from mouse total brain cDNA). TRIM3-RBCC (aa 1-290) was inserted into pJG4-5-vector as *Eco*RI-*Xho*I-fragment, full-length TRIM3 (aa 1-744) and TRIM3-ΔRBCC (aa 291-744) were inserted as *Bgl*II/SalI-fragments into *Bgl*II-*Xho*I sites of a modified pJG4-5-vector (pJG4-5/mod). pJG4-5/mod carrying additional restriction sites was generated by inserting an annealed oligonucleotide-linker via *Eco*RI-*Xho*I-restriction sites:

sense: *aattcgggcccttagatctatcgatccatgggctagctcgcgaccgcggcccgggc*

antisense: tcgagcccgggccgcggtcgcgagctagcccatggatcgatagatctaagggcccg

ShRNA-constructs: for shRNA-mediated knockdown of KIF21B gene expression in cultured hippocampal neurons the pSUPER.neo+GFP-vector (Oligoengine, Seattle, WA) was used. The following oligonucleotides were used for cloning:

KIF21B-shRNA

*sense:* gatccccCCACGATGACTTCAAGTTCttcaagagaGAACTTGAAGTCATCGTGGttttta,

*antisense*:

agcttaaaaaCCACGATGACTTCAAGTTCtctcttgaaGAACTTGAAGTCATCGTGGggg,

scrambled-shRNA:

*sense:* gatccccGCGCGCTTTGTAGGATTCGttcaagagaCGAATCCTACAAAGCGCGCttttta,

*antisense:*

agcttaaaaaGCGCGCTTTGTAGGATTCGtctcttgaaCGAATCCTACAAAGCGCGCggg.

Constructs for FRAP-experiments: To generate mCherry-KIF21B the murine KIF21B-ORF was subcloned by excision from pCMV6-Entry-KIF21B (Origene, Rockville, MD) via *Bgl*II and *Sac*II and insertion into an *Bgl*II - *Sac*II-opened pmCherry-vector. The latter was generated by replacing EGFP from pEGFP-C1-vector (Clontech, Mountain View, CA) with mCherry via *Age*I-*Bgl*II-restriction sites. All constructs were verified by dideoxy sequencing.

### Antibodies

#### Coimmunoprecipitation

rabbit anti-KIF21B (Sigma, Taufkirchen, Germany), rabbit unspecific IgG (Dianova, Hamburg, Germany). *Western blotting*: rabbit anti-KIF21B (1:2,000; Millipore, Schwalbach, Germany) or rabbit anti-KIF21B (1:1,000; Sigma, Taufkirchen, Germany), rabbit anti-TRIM3 (1:2,000; Abcam, Cambridge, UK) or mouse anti-TRIM3 (1:1,00; BD Biosciences, San Jose, CA), anti-GAPDH (1:50,000; Abcam, Cambridge, UK), rabbit anti-Optineurin (1:1,000; Abcam, Cambridge, UK), rabbit anti-Actin (1:5,000; Sigma, Taufkirchen, Germany), chicken anti-NSE (1:2,000; Novus, Cambridge, UK), mouse anti-KHC/KIF5 (clone H2, 1:1,000; Millipore, Schwalbach, Germany), peroxidase-conjugated donkey anti-chicken, donkey anti-rabbit and sheep anti-mouse (1:5,000-1:15,000; Dianova, Hamburg, Germany). *Immunocytochemistry*: rabbit anti-KIF21B (1:100; Sigma, Taufkirchen, Germany), rabbit anti-TRIM3 (1:800; Abcam, Cambridge, UK), mouse anti-MAP2 (clone AP20, 1:300; Sigma, Taufkirchen, Germany), guinea pig anti-GFAP (1:500; Synaptic Systems, Göttingen, Germany), mouse anti-HA-tag (1:100; Roche, Mannheim, Germany), Cy3- or Cy5-conjugated donkey anti-guinea pig, donkey anti-mouse or donkey anti-rabbit (all 1:500; Dianova, Hamburg, Germany), Alexa488 conjugated goat anti-mouse (1:500; Dianova, Hamburg). *Electron* *microscopy* : rabbit anti-TRIM3 (1:400; Abcam, Cambridge, UK).

### Cell culture and transfection

Primary cultures of hippocampal neurons were prepared from neonatal (P0) mice or rats as described [45] with the following modifications: 12 mm coverslips were coated sequentially with poly-L-lysine (1.5 µg/ml in PBS) and laminin (20 µg/ml, Sigma, Taufkirchen, Germany) and then seeded with 110.000 cells/coverslip. Neurons were transfected between DIV5 and DIV11 using a calcium phosphate precipitation protocol (10-20 transfected neurons per coverslip). Per 12 mm coverslip 2 µg of DNA in 25 µl of 250 mm CaCl_2_ were mixed with 25 µl of 2 x HBS (42 mM HEPES, 10 mM KCl, 12 mM dextrose, 274 mM NaCl, 1.5 mM Na_2_PO_4_; pH 7.05). After 15 min incubation at RT the mixture was added to the culture medium. The precipitate was carefully removed after 1 hr and replaced by 600 µl of conditioned medium and 400 µl of fresh Neurobasal medium. For coexpression of HA-tagged TRIM3 together with EGFP 1.5 µg TRIM3-plasmid was mixed with 0.5 µg pEGFP-vector. To induce shRNA-mediated gene-knockdown mouse neurons were transfected at DIV10 with either pSUPER.neo+GFP-KIF21B-shRNA or pSUPER.neo+GFP-scrambled-shRNA and fixed at DIV14.

### Cycloheximide-chase-experiments

To assess the protein half life of KIF21B chase-experiments using the translational inhibitor cycloheximide (CHX) were performed. Hippocampal neurons from wildtype (+/+) or *Trim3*-knockout (-/-) mice were cultured on 12 mm coverslips and treated with 70 µm f.c. CHX (100 mg/ml in DMSO, Sigma, Taufkirchen, Germany) for either 4h, 8h, 24h or 48h. All samples were harvested simultaneously at DIV16 by washing the cells carefully with icecold PBS and adding 125µl cell lysis buffer (1% (v/v) Triton-X-100, 1mM PMSF, 1x Complete Protease Inhibitor Cocktail (Roche, Mannheim, Germany), in PBS). Cells were then scraped off, transferred to a reaction tube and incubated on ice for 45 min. After centrifugation at 1,000 x g for 5 min the supernatant was removed, snapfrozen and stored at -80°C. For analysis same protein amounts of each sample were loaded on a SDS-gel followed by western blotting. Protein concentration was measured using the “BCA Protein Assay Kit” (Pierce Biotechnology, Rockford, IL) prior to boiling probes in SDS sample buffer.

### Immunocytochemistry

Cultured hippocampal neurons were fixed in 4% PFA / 4% sucrose in PBS (12 min), washed in PBS and permeabilized with 0.25% Triton-X-100 in PBS (4 min). After blocking with 1% (w/v) bovine serum albumin in PBS (60 min) cells were incubated with primary antibodies at 4°C overnight, washed in PBS and incubated with secondary antibodies for 1h at RT. Antibodies were diluted in 1% (w/v) bovine serum albumin in PBS. Cells were again washed in PBS and mounted in Aqua Poly Mount (Polysciences, Warrington, PA). When two primary antibodies from the same species (rabbit) were applied following sequential staining protocol was used: cultured hippocampal neurons were fixed and permeabilized as described above. Cells were blocked in 2.5% (v/v) donkey serum in PBS, the same solution was used for antibody dilution. After incubation with the first primary antibody and extensive washes a monovalent secondary antibody was applied in high concentration (1:125) for 1h at RT. After extensive washes cells were then incubated with the second primary antibody, followed by washes and incubation of the second secondary antibody in normal concentration (1:500). Following control stainings were done: to assess for binding of the 2nd secondary antibody to the 1st primary antibody, cells were stained as described above but instead of the 2nd primary antibody blocking solution only was applied. To assess for binding of the 1st secondary antibody to the 2nd primary antibody, blocking solution only was applied instead of 1st primary antibody.

### Electron microscopy

For postembedding immunogold-stainings adult mice were anaesthetized and perfused with 4% PFA and 0.1% (w/v) glutaraldehyde in 0.1 M phosphate buffer. 1mm^3^ pieces were taken from the hippocampus, embedded with 12% gelatine in PBS at 37 °C for 15 min and cryoprotected overnight by immersion in 2.3 M sucrose in PBS. Tissue pieces were then mounted on specimen holders and snapfrozen in liquid nitrogen. Thereafter ultrathin sections of 80 nm were cut at -120°C using an ultramicrotom (Reichert, Seelfeld, Germany) and mounted in coppernets covered with pioloform. Prior to immunogold-stainings specimens were incubated for 20 min at 40 °C to dissolve away the gelatine and were then blocked in 50 mM glycine in PBS followed by blocking in AURION blocking solution (Wageningen, Netherlands). All following antibody dilutions and blocking steps were carried out in 0.1% (v/v) AURION-BSA-c in PBS. After blocking sections were washed (2x 5 min) and incubated with primary antibody for 1 h at RT. Sections were again extensively washed (6x 5 min) and incubated with secondary antibody for 45 min at RT. After washing 10 nm Protein A-Gold particles (G. Posthuma, University of Utrecht, Netherlands) were applied according to the manufacturer’s protocol. Specimens were then washed, fixed for 5 min in 1% glutaraldehyde in PBS and washed in H_2_O. Finally ultrathin sections were contrasted by incubation with 1.8% (w/v) methylcellulose and 0.4% (w/v) uranylacetate in H_2_O for 2 min on ice and dried on filter paper. Ultrathin sections were examined with a Zeiss EM 902 (Zeiss, Göttingen, Germany). Preembedding immunoelectron microscopy with diaminobenzidine (DAB) was performed according to [37].

### Coimmunoprecipitation and differential centrifugation

All steps were carried out at 4 °C. 30 µl “Dynabeads Protein G” (Life Technologies, Darmstadt, Germany) were washed in IP-buffer (50 mM Tris (pH 7.5), 150 mM NaCl, 5 mM MgCl_2_) and incubated overnight with 2 µg specific antibody or control IgG. After washing with IM-Ac-buffer (20 mM HEPES, 100 mM K-Acetate, 40 mM KCl, 5 mM EGTA, 5 mM MgCl_2_; pH 7.2) antibody-coupled beads were incubated for 30 min with brain lysates. Beads were then extensively washed with modified RIPA-buffer (50 mM Tris (pH 7.5), 150 mM NaCl, 1 mM EDTA, 1% (v/v) NP40, 0.25% (w/v) sodiumdeoxycholate, 0.5% (v/v) Triton-X-100), boiled in SDS sample buffer and analyzed by western blotting. Brain lysates were obtained by differential centrifugation from whole rat brains (postnatal day 10) as described in Saito et al. 1997. For coimmunoprecipitation P4 (400,000 x g) pellets resuspended in IM-Ac-buffer (supplemented with 1x Complete Protease Inhibitor Cocktail (Roche, Mannheim, Germany), 1mM PMSF, 5 mM DTT and 2 mM MgATP) were used. Prior to antibody-coupling P4-suspensions were incubated with 1% f.c. Triton-X-100 and precleared with uncoupled Dynabeads. To compare protein contents in hippocampi from wildtype (+/+) and *Trim3*-knockout (-/-) mice animals of each genotype were sacrificed at postnatal day 15. The hippocampi were homogenized in ice cold supplemented IM-Ac-buffer and centrifuged at 1,000xg for 10 min at 4°C. The supernatant (S1) was aliquoted, snap frozen and stored at -80°C. For analysis same protein amounts of wildtype (+/+) and knockout (-/-) samples were loaded on a SDS-gel followed by western blotting. Protein concentration was measured using the “Bio-Rad Protein Assay Dye Reagent Concentrate” (Bio-Rad, Munich, Germany) prior to boiling probes in SDS sample buffer.

### Generation and genotyping of *Trim3*-knockout-mice

#### Trim3 targeting construct

A 9.6 kb *Sca*I*-Sal*I mouse genomic DNA fragment containing exons 3-9 of the *Trim3* gene was subcloned in pACYC184. A neomycin resistance cassette (Neo^r^) was cloned in the forward direction into the SwaI restriction site upstream of exon 3. Neo^r^ was flanked on both sides by frt sites and on the 3’ end also by a loxP site. A second loxP site containing a downstream *Bgl*II restriction site was cloned into the *Sph*I restriction site between exons 5 and 6. The resulting *Trim3* targeting construct was linearized and used to electroporate mouse embryonic stem (ES) cells.

#### Electroporation and selection of ES cells

Linearized *Trim3* targeting construct (100 µg) was ethanol precipitated, dissolved in 300 µl PBS and mixed with 500 µl PBS containing 10,000,000 ES cells of the E14.1 line derived from 129P2/OlaHsd mice. ES cells were electroporated using a Biorad (Hercules, CA) Gene Pulser 165-2087 (280 V, 500 µF, 6.0 msec time constant). After 5 min incubation at RT, electroporated cells were resuspended in 50 ml Dulbecco’s minimal essential medium supplemented with 10% fetal calf serum, non-essential amino acids, 0.1 mM β-mercaptoethanol, and 1,000 U/ml leukemia inhibitory factor (ESGRO-LIF; Millipore, Billarica, MA). Cells were seeded into 5 gelatin-coated culture dishes (diameter: 10 cm) containing a feeder layer of mouse embryonic fibroblast that were mitotically inactivated using Mitomycin C (Sigma-Aldrich, St. Louis, MO; 10 µg/ml for 2 h). One day after seeding, the medium was replaced by fresh medium containing 400 µg/ml G418. Medium was subsequently refreshed every 2-3 days. G418-resistant colonies were picked after 3-6 days, trypsinized and expanded in 96-well plates. Genomic DNA was isolated using the Wizard® Genomic DNA Purification Kit (Promega, Madison, WI) according to the manufacture’s instructions. Individual clones (576 in total) were screened for insertion of the neomycin gene into the *Trim3* genomic locus by use of a nested PCR with two forward primers, primer a (GTGTTTCTTCAATATACACCCATG) and primer b (TGGAATATCCTGAGTATGTGAG), located upstream of the *Trim3* targeting construct, and two reverse primers, primer 1 (CCTACCCGGTAGAATTGGC) and primer 2 (CAGACTGCCTTGGGAAAAGCG), located inside the neomycin gene. PCRs were performed using Turbo *Pfu* polymerase (Stratagene, La Jolla, CA). Of the first PCR (primers a and 2; 40 cycles), 1 µl was used as a template for the second, nested PCR (primers b and 1; 25 cycles). ES cell clones yielding the expected PCR product of ~1.7 kb were then checked for the presence of the second loxP site using primer c (CCCCATCCACACGTGTGTC) and primer 3 (GTCTAACTGTGTCTGCAACAC). To confirm homologous recombination of the endogenous *Trim3* gene, two PCRs were performed combining primers outside and inside the *Trim3* targeting construct and using the Expand High Fidelity PCR System (Roche, Basel, Switzerland). Primers b and 3 were used to check homologous integration of the 5’ end of the targeting construct and primers c and 4 (CTGACTGGGTTGAAGGCTCTC) to check homologous integration of the 3’ end. Both PCR products were digested with Bgl*II* in order to confirm their identity. Two positive clones (1C12 and 1E9) were expanded and injected into blastocysts derived from C57Bl/6 mice, yielding eight chimeric animals. After germline transmission of the mutant Trim3 gene from clone 1E9, a colony of heterozygous *Trim3* ^lox/+^ animals was maintained by backcrossing to inbred C57Bl/6 mice.

#### Generation of Trim3 -/- mice

homozygous Trim3^lox/lox^ animals were crossed with 129Cre mice expressing Cre-recombinase under the control of a human cytomegalovirus minimal promoter [30]. Heterozygous F1 mice (Trim3^+/l-^) were selected and crossed with each other to generate homozygous knockout animals (Trim3^-/-^).

#### Genotyping of Trim3 knockout mice

For genotyping tail biopsies were lysed overnight at 55°C in 200 µl tail lysis buffer (2.5 mM EDTA, 50 mM KCl, 10 mM Tris-HCl (pH 8.5), 0.45% (v/v) Tween 20, 0.45% (v/v) NP40, 1mg/ml f.c. Proteinase K (Roche, Mannheim, Germany)). After heat inactivation at 95 °C for 15 min this genomic DNA solution was used for genotyping PCR. Following primers were used: A. TRIM3-Int4-FW (ccccatccacacgtgtgtc), B. TRIM3-Int4-RV (ctactgccaatgtgctcctg 3’), C. *TRIM3*-knockout-FW (gtgtgccaccatcagtgagatac). By combining primers A+B and C+B wildtype (+/+) and knockout (-/-) gene loci could be differentiated as follows: A+B wildtype (+/+) = 179 bp; A+B knockout (-/-) = no PCR product; C+B wildtype (+/+) = ca. 2.5 kb; C+B knockout (-/-) = 462 bp).

### Image Processing and Statistical Analysis of Data acquired by Western Blotting and Immunofluorescence

Western blot signals were acquired as TIFF-files using the CCD-camera-based imaging system “Intas ChemoCam” (Intas, Göttingen, Germany) and raw data signals subsequently analyzed using the analysis software ImageJ, version 1.42q (National institutes of health, Bethesda, MD). To evaluate relative Western blot signal intensities signal intensities were normalized to loading control signals (Actin or NSE). Statistical analysis was performed in Microsoft Excel. The student’s t-test was used to assess statistical significance with following definitions: * = p ≤ 0.05; ** = p ≤ 0.01; ***= p ≤ 0.001. All values from quantitative data are reported as mean ±SEM from n independent experiments. Error bars represent the SEM.

Fluorescence imaging was carried out with an upright Olympus FluoView FV1000 (Olympus, Hamburg, Germany) laser scanning confocal microscope using 63x objective and sequential channel recording mode. Confocal images from multiple individual cells used for statistical analysis were obtained as overlay TIFF-files using identical photomultiplier values throughout each experiment and genotypes. Experiments were at least replicated three times. Images were further analyzed using MetaMorph 6.3r7 (Molecular Devices, Sunnyvale, CA). TIFF-files were first separated in single channel images using the “color separate” function followed by threshold adjustment using the “threshold images” function. Identical threshold levels were applied throughout an experiment. To measure fluorescent intensities of somatic regions (without nucleus) the region of interest (ROI) was selected on overlay images using the “trace region” function and subsequently transferred to single channel images using the “transfer region” function. Mean fluorescent intensities (total grey value/area) were then retrieved for each ROI/channel using the “show region statistics” function. Statistical analysis was again performed with Microsoft Excel as described above.

### Live cell imaging

For live cell imaging hippocampal neurons were plated on glass bottom dishes coated with 5µg/ml poly-D-lysine. Cells were transfected with mCherry-KIF21B between DIV 5 (-/-) and DIV 10 (+/+) and imaged after 16hrs. For time-lapse video microscopy, a spinning disk confocal imaging system, equipped with an inverse Nikon Ti-E microscope and a 2D-VisiFRAP Galvo System or an Axiovert 200M (Zeiss, Göttingen, Germany) equipped with a CCD camera was used. During recording cells were temperature controlled (37°C). The mobility and speed of vesicles was analysed manually using Metamorph software (Molecular Devices, Sunnyvale, CA), as described [46].

### FRAP-experiments

To investigate the recovery coefficient of mCherry-tagged KIF21B and mCherry alone in dendrites of wildtype (+/+) and *Trim3*-knockout (-/-) hippocampal neurons cells cultured on 12 mm coverslips were transfected at DIV10 with the corresponding plasmids. After 24h expression dendritic regions of 20 µm length were bleached. For this an upright Olympus FV1000 confocal microscope (Olympus, Göttingen, Germany) equipped with a second scanner for simultaneous bleaching and a 405 nm UV-laser diode (Melles Griot, Irvine, CA) was used. The pinhole was fully opened and a 20 µm diameter circle was bleached with the 405nm laser and 85% laser intensity using the Tornado scanning function. Imaging of the bleached region using the 535 nm laser at 23% intensity started directly when bleaching ended (bleaching time of 0.5 sec for mCherry and 4 sec for mCherry-KIF21B). For mCherry alone images were taken every second for 300 seconds. For mCherry-KIF21B images were taken every 5 sec for 500 seconds. To calculate the recovery coefficient (D_app_), signals of the complete region of the bleached area (20 µm) containing the entire width of the dendrite were averaged. The background obtained from regions outside the dendrite was subtracted for each frame. To compensate for bleaching, intensities of fluorescent areas that were not part of the bleached dendrite were measured and the values corrected accordingly for each frame.

### Cell extraction

Hippocampal neurons derived from wildtype (+/+) and *Trim3*-knockout (-/-) mice were plated in 24-well plates. At DIV20-23, cells were washed with prewarmed HEM-buffer (80 mM HEPES (pH 6.9), 1 mM EGTA, 2mM MgCl_2_). 100 µl HEM containing 0.2% Triton-X-100 was added for 1 min. The supernatant was subsequently removed and transferred into a separate reaction tube. 100 µl lysis buffer (50mM TrisHCl (pH7.4), 10% Glycerol, 1% NP40, 5mM DTT, 1mM NaEGTA, 20mM NaF, 1mM Vanadate, 150mM NaCl, 1mM PMSF, Proteininhibitor cocktail, 5µM CHAPS) were added to the remaining pellet fraction containing the cytoskeleton (24-well plate). The pellet was subsequently harvested with a cell scraper. Equal volumes of supernatant and pellet fractions were further subjected to western blotting. For statistical analysis, the amount of KIF21B in the supernatant (%) was determined relative to the amount of KIF21B in the corresponding pellet (KIF21B pellet signal intensity set to 100%). The students’s t-test was used for statistical analysis of KIF21B supernatant content across the genotypes (wildtype (+/+) values set to 1).

## Supporting Information

Figure S1 **Controls**.
(A) Antibody specificity control. Absence of KIF21B immunoreactive signals in cultured hippocampal neurons after induction of shRNA-mediated knockdown of KIF21B expression. Cells were transfected with plasmids encoding scrambled or KIF21B-shRNA, respectively, and coexpressing EGFP under an independent promotor for transfection control. (Scale bars: 20 µm.) (B) Control experiments to exclude cross talk in sequential immunostainings using rabbit KIF21B- and rabbit TRIM3- antibodies. Cy5-secondary antibody did not bind to Cy3-bound KIF21B-antibody, confirming Cy3-saturation on KIF21B-binding sites (B1). Cy3-secondary antibody did not bind to Cy5-bound TRIM3-antibody, confirming complete Cy3-wash-off before application of TRIM3-primary antibody (B2). Cy5 is shown in false color for better visualization. Scale bars: 10 µm. (C) Genomic PCRs confirming homologous recombination and complete integration of the *Trim3* targeting construct in ES cell clone 1E9. Primers c-3 confirm integration of the second loxP site. Primers b-3 confirm integration of the Neo^r^ cassette and exons 3-5. Digestion of this product with *Bgl*II confirms its identity; the smaller restriction fragment (1643 bp) is observed due to the presence of a *Bgl*II site in the Neo^r^ cassette. Primers c-4 confirm integration of the second loxP site and exons 6-9. Digestion of this product with *Bgl*II confirms its identity; two unique *Bgl*II restriction fragments (1374 bp and 135 bp) are observed due to the presence of a *Bgl*II site immediately downstream of the loxP site. (D-F) Analysis of mCherry-KIF21B mobility in TRIM3 depleted neurons. (D) Particles were analysed with an image acquisition rate: 1 image/2 sec. (E) The corresponding kymograph represents a region of 45µm. (F) Schematic representation of a mobile (arrow in D) and of stationary particles. antero: anterograde direction, retro: retrograde direction.
(TIF)

Movie S1 **Movie of data shown in Figure 5A.**
(AVI)
