# Supplementary figures and images for: TRIM3 Regulates the Motility of the Kinesin Motor Protein KIF21B
Source: PLoS One. 2013 Sep 24;8(9):e75603. doi: 10.1371/journal.pone.0075603 (PMC3782429; doi:10.1371/journal.pone.0075603)

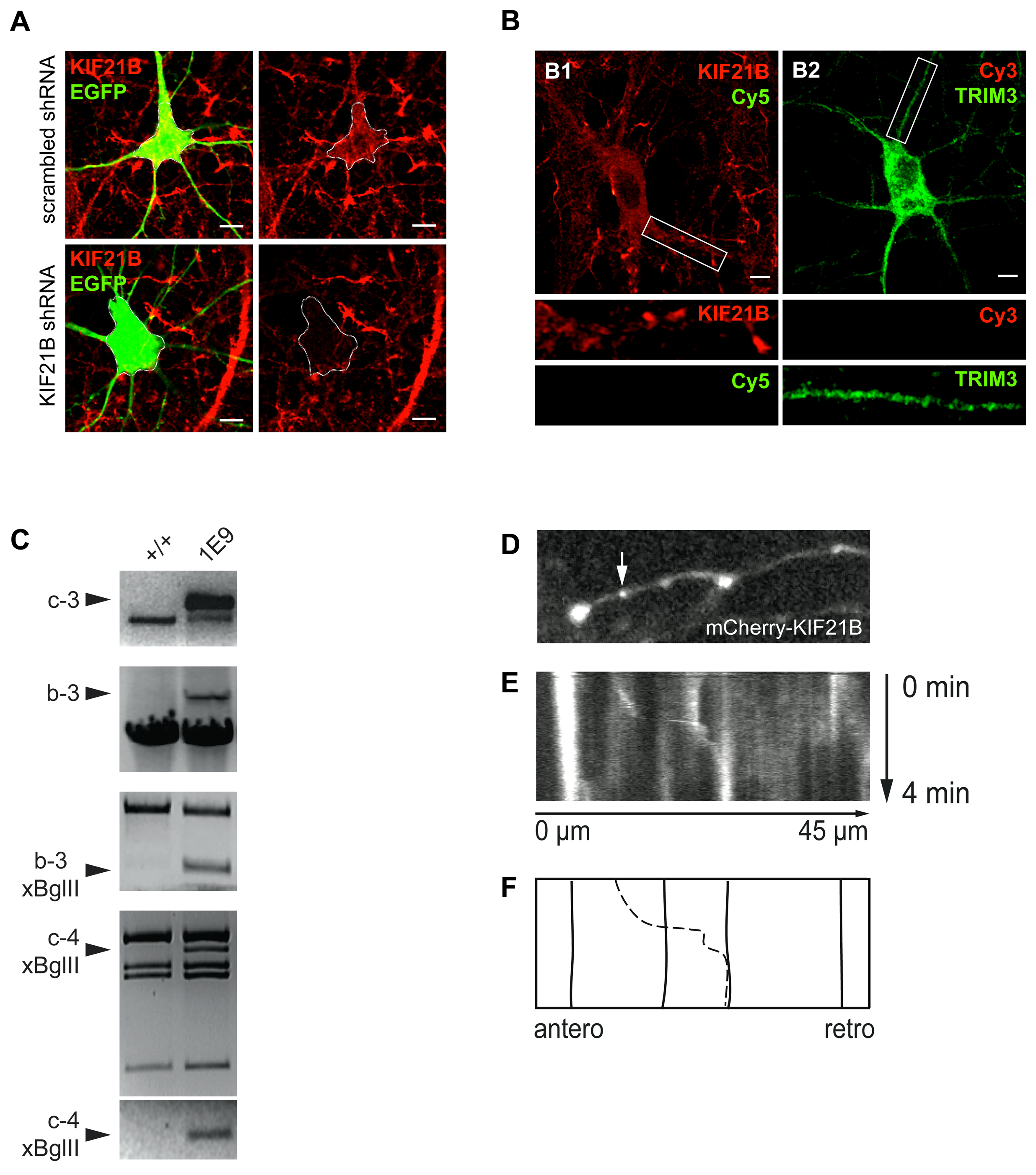

Supplement: Figure S1 — Controls. (A) Antibody specificity control. Absence of KIF21B immunoreactive signals in cultured hippocampal neurons after induction of shRNA-mediated knockdown of KIF21B expression. Cells were transfected with plasmids encoding scrambled or KIF21B-shRNA, respectively, and coexpressing EGFP under an independent promotor for transfection control. (Scale bars: 20 µm.) (B) Control experiments to exclude cross talk in sequential immunostainings using rabbit KIF21B- and rabbit TRIM3- antibodies. Cy5-secondary antibody did not bind to Cy3-bound KIF21B-antibody, confirming Cy3-saturation on KIF21B-binding sites (B1). Cy3-secondary antibody did not bind to Cy5-bound TRIM3-antibody, confirming complete Cy3-wash-off before application of TRIM3-primary antibody (B2). Cy5 is shown in false color for better visualization. Scale bars: 10 µm. (C) Genomic PCRs confirming homologous recombination and complete integration of the Trim3 targeting construct in ES cell clone 1E9. Primers c-3 confirm integration of the second loxP site. Primers b-3 confirm integration of the Neor cassette and exons 3-5. Digestion of this product with BglII confirms its identity; the smaller restriction fragment (1643 bp) is observed due to the presence of a BglII site in the Neor cassette. Primers c-4 confirm integration of the second loxP site and exons 6-9. Digestion of this product with BglII confirms its identity; two unique BglII restriction fragments (1374 bp and 135 bp) are observed due to the presence of a BglII site immediately downstream of the loxP site. (D-F) Analysis of mCherry-KIF21B mobility in TRIM3 depleted neurons. (D) Particles were analysed with an image acquisition rate: 1 image/2 sec. (E) The corresponding kymograph represents a region of 45µm. (F) Schematic representation of a mobile (arrow in D) and of stationary particles. antero: anterograde direction, retro: retrograde direction. (TIF) [file pone.0075603.s001.tif]
